# Supplementary material for: The causal relationship between the human gut microbiota and pyogenic arthritis: a Mendelian randomization study
Source: Front Cell Infect Microbiol. 2024 Nov 26;14:1452480. doi: 10.3389/fcimb.2024.1452480 (PMC11629706; doi:10.3389/fcimb.2024.1452480)
Supplement: Supplementary file 1 [file DataSheet1.zip › mendelian test/ebi-a-GCST90027479.csv_leave_one_out.pdf]

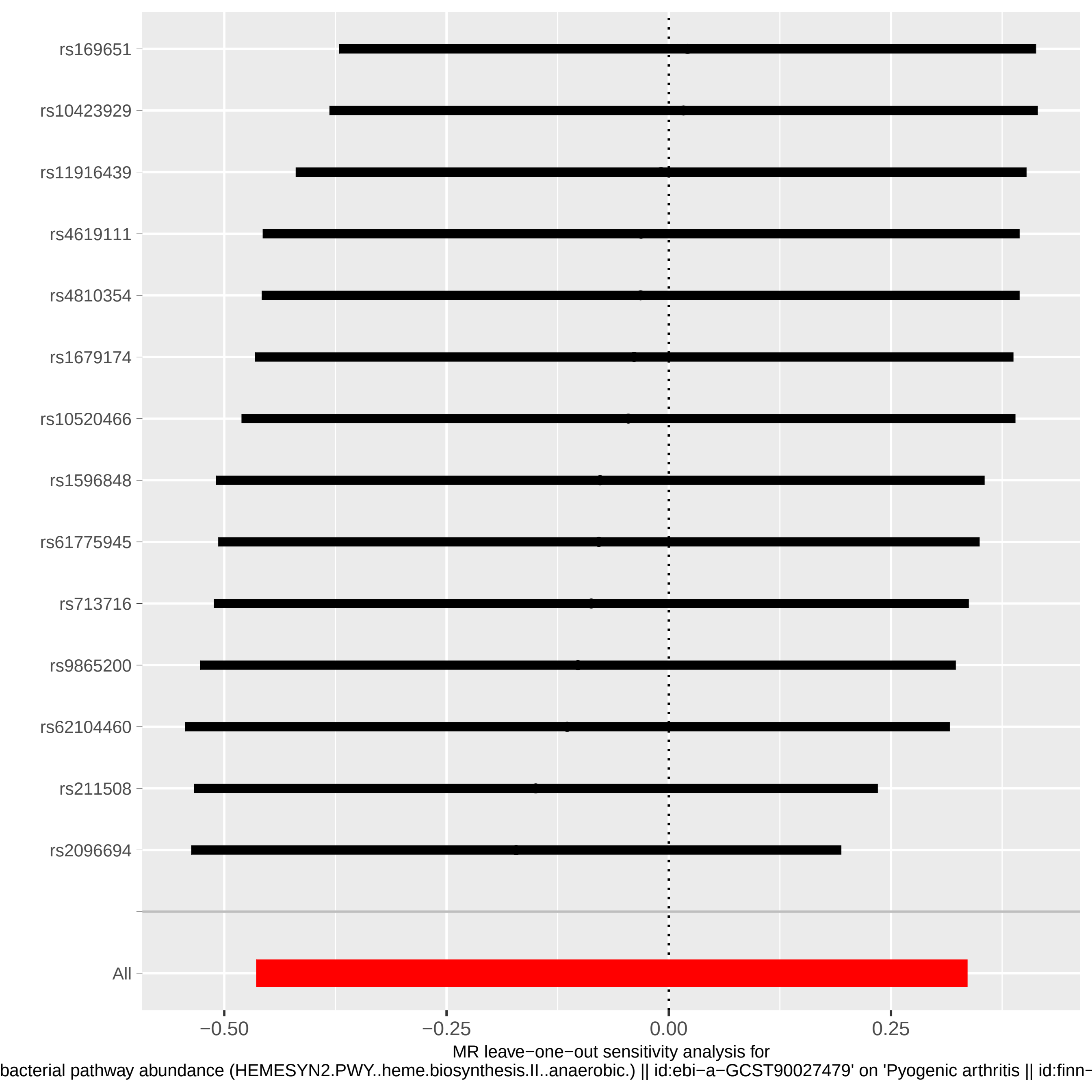
rs169651
rs10423929
rs11916439
rs4619111
rs4810354
rs1679174
rs10520466
rs1596848
rs61775945
rs713716
rs9865200
rs62104460
rs211508
rs2096694
All
−0.50
−0.25
0.00
0.25
MR leave−one−out sensitivity analysis for
bacterial pathway abundance (HEMESYN2.PWY..heme.biosynthesis.II..anaerobic.) || id:ebi−a−GCST90027479' on 'Pyogenic arthritis || id:finn−
